# More Than Epineurium Deep: Characterizing Peripheral Nerve Damage Using High-Resolution Micro-Computed Tomography for Simulated Peripheral Nerve Lacerations

**DOI:** 10.1016/j.jhsg.2025.100833

**Published:** 2025-09-18

**Authors:** Rasa Zhukauskas, Brandon S. Smetana, Adam B. Strohl, Sunishka M. Wimalawansa, Eitan Melamed, Amy M. Moore, Fraser J. Leversedge, Youssra Marjoua, Bauback Safa

**Affiliations:** ∗ Axogen Corporation, Alachua, FL; † Indiana Hand to Shoulder Center, Indianapolis, IN; ‡ Philadelphia Hand to Shoulder Center, Philadelphia, PA; § Boonshoft School of Medicine/Miami Valley Hospital, Department of Orthopaedics, Division of Plastic Surgery, Wright State University, Dayton, OH; ‖ NYC Health + Hospitals Elmhurst, Queens, NY; ¶ The Ohio State University College of Medicine, Department of Plastic and Reconstructive Surgery, Columbus, OH; ∗∗ University of Colorado School of Medicine, Department of Orthopedic Surgery, Aurora, CO; †† The Cleveland Clinic, Department of Orthopaedic Surgery, Cleveland, OH; ‡‡ The Buncke Clinic, San Francisco, CA

**Keywords:** Damaged nerve, Lacerated nerve, Micro-CT, Nerve injury, Nerve trauma

## Abstract

**Purpose:**

Nerve damage because of acute traumatic lacerations is challenging to assess and is typically evaluated using loupes or an operating microscope. However, defining the zone of injury clinically is limited to evaluating the epineurium and/or transected nerve ends for visible injury, with tactile changes corresponding with nerve damage not evident in the acute setting. During surgical nerve repair or reconstruction, adequate debridement of the damaged tissue is essential, as fascicular health influences the regenerative potential of the nerve. This study used a novel high-resolution imaging method to characterize the extent of nerve damage resulting from 3 common mechanisms of traumatic lacerations.

**Methods:**

Twelve human upper extremity cadaveric specimens were used to simulate common injuries using a knife, broken glass, or table saw in flexor tendon zones II or V. The distance of nerve damage measured from the transected end was visually estimated by experienced peripheral nerve surgeons under loupe magnification. The length of nerve damage was measured radiographically using micro-computed tomography and then compared with visually estimated damage.

**Results:**

Radiographic image analysis revealed fascicular disruption extending proximally and distally from the transection, which was underestimated by visual assessment 9.5 mm on average in knife injuries, 7.8 mm in broken glass injuries, and 12.1 mm in table saw injuries. The extent of radiographic damage was similar in proximal and distal nerves, and in knife and broken glass lacerations, but most extensive in table saw lacerations.

**Conclusions:**

Nerve damage was greatest in table saw lacerations. Radiographic imaging showed fascicular damage extending beyond the surgeon’s visual assessment of epineural damage, indicating that the internal damage to nerves from traumatic lacerations was underappreciated by surgeons. The impact this underestimated damage has on regenerative potential of an injured nerve requires further investigation.

**Type of study/Level of evidence:**

Diagnostic V.

Severe high-energy nerve injuries can lead to structural disorganization within the nerve, well beyond the point of rupture.^1^ Therefore, understanding the extent of nerve damage is important during the surgical reconstruction of traumatic nerve injuries to ensure that nerve damage has been adequately identified and resected to optimize sensorimotor outcomes and prevent neuroma formation.^2^, ^3^, ^4^, ^5^ Evaluation of nerve damage in the clinical setting is limited because of resolution of current intraoperative diagnostic methods, primarily loupes or a surgical microscope. Surgeons often rely on touch (a healthy nerve feels soft and pliable, whereas scar tissue tends to feel more firm^2^) or visual characteristics of the cut nerve end to determine the extent of damage. However, tactile assessment of nerve damage extent is marginally useful in the acute setting, where surgeons are most reliant on visible signs of pathology.

Clinically, a conservative nerve tissue preserving approach is often used when resecting an injured nerve to limit the potential nerve gap between ends; however, this may lead to inadequate resection of damaged tissue, thereby limiting nerve regeneration and causing poor outcomes. As an example, Jain et al^6^ reported that 32% of patients who underwent symptomatic neuroma resection required treatment because of a failed direct nerve repair. These poor outcomes may be exacerbated by the inability to assess nonvisible nerve damage, which may lead to scar within the nerve.

The internal structure of nerve may be visualized using micro-computed tomography (micro-CT), which can acquire high-resolution 3-dimensional images.^7^^,^^8^ Micro-CT has been used to visualize the fascicular morphology along the length of a nerve including manually segmenting and tracing fascicles^9^ and delineating organotypic fascicular connectivity.^10^ The goal of our study was to compare the extent of morphological peripheral nerve damage caused by knife, glass, and saw lacerations. Leveraging micro-CT imaging may expand our understanding of the extent of nerve damage from these common lacerations. As a comparator, injuries were measured visually by expert nerve surgeons under loupe magnification. We hypothesized that using a novel high-resolution micro-CT imaging would reveal a greater extent of damage than using loupe evaluation.

## Materials and Methods

**Figure 1:**
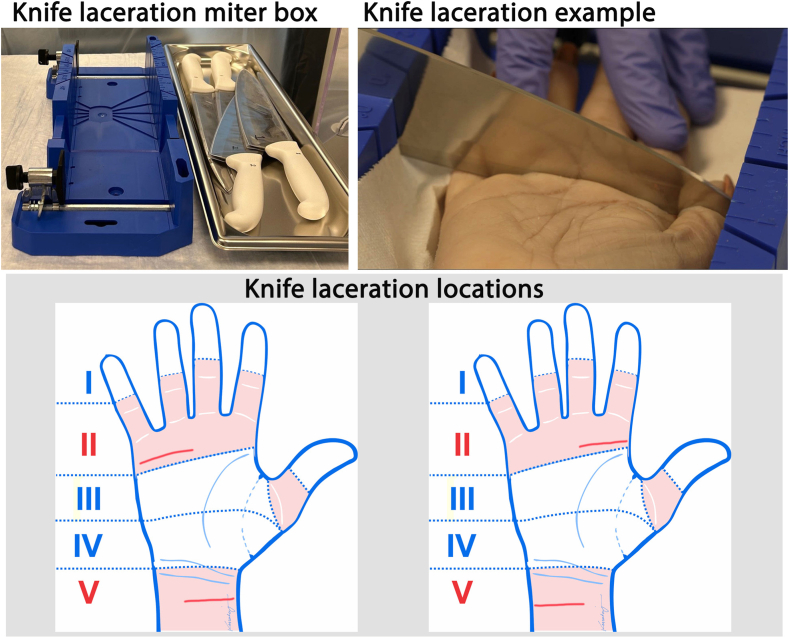
Knife laceration miter box, example of knife laceration creation, and locations of knife lacerations (red lines) in anatomical zones II or V.

Twelve upper extremity specimens were used to create injuries in various anatomical regions. Cadaveric specimens were from donors aged between 26 and 70 years with a body mass index between 21 and 30 kg/m^2^, consented for research (Table 1). Lacerations were created in designated flexor tendon anatomical zones, determined by the most common laceration location and biological sex.^11^, ^12^, ^13^, ^14^, ^15^, ^16^, ^17^ Four specimens were used for each mechanism of injury, with 1 mechanism of injury in each specimen. Resting tension was simulated by suturing the proximal end of the specimen closed, just below the humeral head, and a tightly secured tourniquet was placed just proximal to the elbow. Each type of injury was induced consistently across specimens by a single technician. All knife lacerations were created using an unused smooth, nonserrated kitchen knife. Knife sharpness was measured using Sharp PT50A testing device prior to use (ranging 216 gf–256 gf). Each specimen was secured in a miter box, and the knife was passed through the miter box slots perpendicular to the longitudinal axis of the specimen. The knife was held in the technician’s dominant hand and moved in a forceful downward motion. Each specimen received 2 lacerations with 1 knife: 1 in anatomical zone II and 1 in zone V (Fig. 1).

**Table 1:** Specimen Injuries and Characteristics

Mechanism of Injury	Sex (#)	Median Age, Range (y)	Median Height, Range (Inches)	Median BMI, Range
Saw	4 Males	53, 52–61	72, 70–72	24.6, 24.4–26.4
Glass	2 Males 2 Females	53.5, 46–62	67, 66–69	25.2, 21.5–29.4
Knife	4 Females	61.5, 50–68	63.5, 62–69	26.8, 25.9–27.3

**Figure 2:**
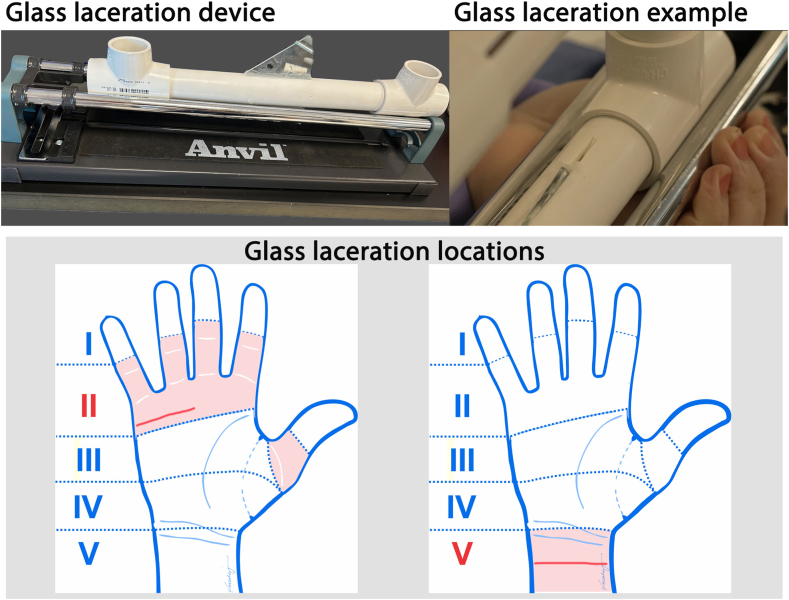
Glass laceration device, example of glass laceration, and broken glass laceration locations, identified by red lines in anatomical zones II or V.

Broken glass lacerations were introduced using a new straight piece of 3/32-inch-thick broken pane glass embedded into a piece of a 2-inch PVC pipe, secured in a tile cutter blade slider. Each specimen was placed under the blade slider with the longitudinal axis perpendicular to the blade slider axis, and the slider was then pushed over the most common locations, anatomical zones II and V (Fig. 2).

**Figure 3:**
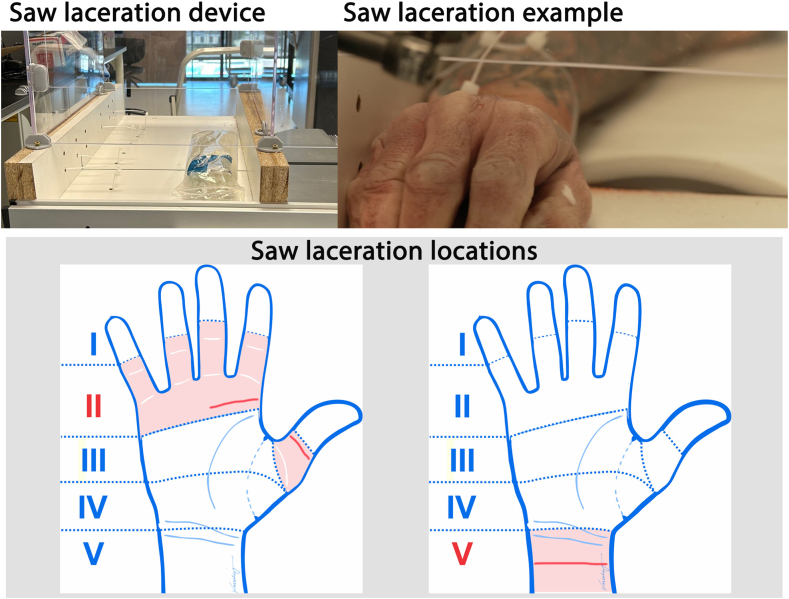
Saw laceration device, example of saw laceration device, and table saw laceration locations, identified by red lines in anatomical zones II or V.

Saw injuries were created using an electric table saw (RIDGID 15 Amp 10-inch portable corded jobsite table saw with folding stand). Each specimen was placed into a custom-made frame and secured with zip ties such that the injuries were oriented perpendicular to the longitudinal axis of the specimen. The blade was powered to maximum speed before creating the injury in anatomical zones II and V (Fig. 3).

Lacerations were evaluated by 8 surgeons experienced in peripheral nerve injury assessments. Laceration sites were surgically dissected and evaluated for injury under loupe magnification (ranging from 3.5× to 4.5×). Each surgeon determined the length of nerve damage measured from the transected ends and the corresponding estimated length of nerve trimming needed to reach healthy tissue proximally and distally (see Table S1, available online on the *Journal’s* website at www.jhandsurg.org. for manuscript definitions). Following visual assessment, injured nerves were mobilized and photographed using a high-resolution camera. The postinjury in situ gap length, between the distal and proximal nerve ends, after resection (or estimated debridement distance) was measured by using high-resolution photographs and an embedded ruler. This measured gap length was added to the measured length of nerve damage to estimate the total gap length after debridement of unhealthy tissue.

Samples of injured and uninjured nerves were collected simultaneously from cadaveric specimens, prepared, and analyzed as previously described.^7^^,^^8^ Nerve samples were stored in 10% neutral-buffered formalin. Before imaging, samples were rinsed in 1× phosphate-buffered saline and exposed to 1.5% buffered Lugol (Heiltropfen, pharma grade, cat # 12298-68-9) for 9–10 days.^7^ Micro-CT imaging was performed using SkyScan 1272 CMOS edition scanning system at Micro Photonics using previously developed system settings for all samples.

During micro-CT imaging, each sample was fixed to a carousel base located between the X-ray source and the detector, which was then rotated 360° around its axis to capture 2-dimensional projection images. The radio-density of fascicular structures was enhanced using an iodine-based contrast agent,^7^ which has an affinity to lipid-containing anatomical structures such as adipose tissue and myelin. An automated segmentation, based on gray-scale values representing fascicles, was performed with Bruker CTvox 3D Suite software to identify fascicular structures. Adipose tissue and other nonfascicular tissues were removed from the images. Thickness measurements of nerve structures were recorded 5 μm apart and output into 2-dimensional color-coded images. Images were reconstructed to create 3-dimensional renderings and videos for each sample (see Supplementary data, available online on the *Journal’s* website at www.jhandsurg.org. for example videos of 3-dimensional renderings of videos in Videos S2 and S3). Radiographs were analyzed using Dragonfly 3D World software (v 2024.1, Comet Technologies).

**Figure 4:**
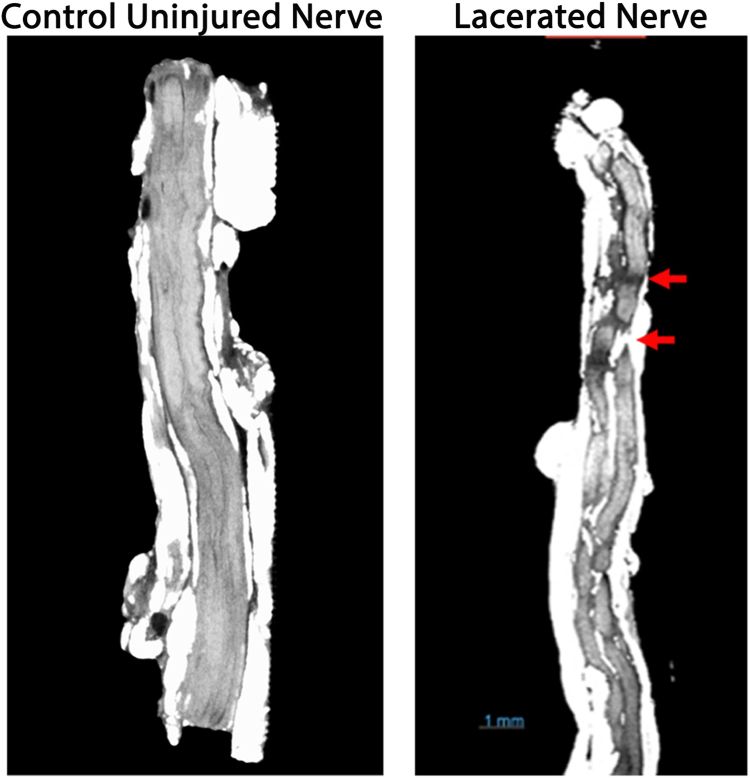
Representative radiographs. Control uninjured nerve samples were used as the benchmark for nerve imaging of lacerated nerves. Red arrows indicate areas of “skip” lesions, indicating damage.

Nerve cross-section radiographs were used to identify epineural damage (see Supplementary data, available online on the *Journal’s* website at www.jhandsurg.org. for examle Video S4). Epineural damage was recorded as the length measured from the lacerated nerve end to where intact epineurium was circumferentially identified. Fascicular damage was evaluated using longitudinal and cross-sections. The fascicular damage was identified by radio-density changes along the length of the sample from the lacerated end through the adjacent tissue until the area of consistent radiopacity, indicative of healthy tissue, was identified. Decreases in radiopacity were attributed to trauma from lacerations, as they were not seen in control uninjured nerve samples (Fig. 4).^8^

**Figure 5:**
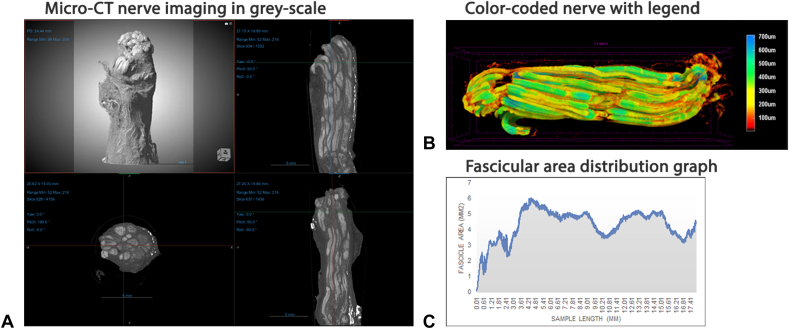
**A** Micro-CT gray-scale nerve images. **B** Color-coded nerve and color code legend indicating of fascicle size in micrometer. **C** Output of fascicular area distribution, where low radio-density is indicated by a decreased area.

**Figure 6:**
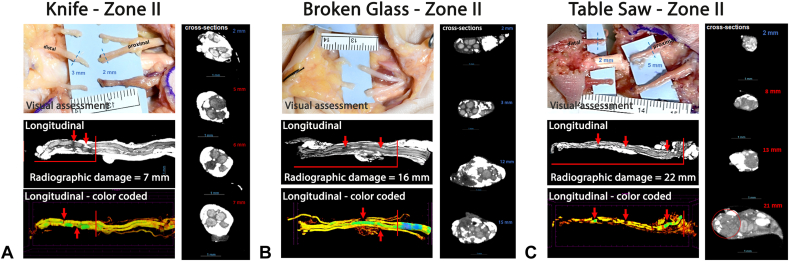
Representative images of **A** knife, **B** broken glass, and **C** table saw injuries. Images include visually estimated nerve damage (left top), original radiographic fascicular nerve damage measurement (left middle), color-coded radiographic fascicular nerve damage measurement (left bottom), and radiographic nerve cross-section for each laceration mechanism (right). Red arrows indicate areas of “skip” lesions.

Fascicular damage was measured from the tip of the lacerated nerve end to the furthest level with notable decreases in radio-density including any “skip” lesions identified at a distance from the injured nerve face. Additionally, these measurements output a plot of fascicular area distribution along the length of each sample (Fig. 5A–C). The senior authors confirmed length of disrupted nerve. R.Z. confirmed damage by manually comparing software-generated fascicular area distribution within cross-section segmentation images of each sample and visual interpretation of radiographic changes present in the 3-dimensional renderings of each sample, which was secondarily verified by a qualified radiologist (Fig. 6A–C).

An analysis of the differences in length of nerve damage was performed between visually estimated measurements compared with radiographic measurements, radiographic measurements of the proximal nerve compared with the distal nerve, radiographic measurements of injuries in anatomical zone II compared with zone V, and radiographic measurements of nerves injured with a knife versus broken glass versus table saw. Selected continuous data were expressed as group mean ± SD and 95% confidence interval (95% CI). This study attained 64 measurements from surgeon visual assessments and 44 measurements from micro-CT analysis. Surgeons visually assessed the damage of both zone II palmar digital nerves, which resulted in total of 64 measurements. Micro-CT imaging was performed on only distal and proximal samples of 1 lacerated palmar digital nerve from each specimen. A minimum difference of 5 mm was a practically significant difference between surgeon visual assessments and radiographic micro-CT measurements of nerve damage. Numerical data sets were evaluated for normality using D’Agosino and Pearson test. A one-way ANOVA test was conducted for data sets with normal distribution; otherwise, a Kruskal-Wallis test was performed. A *P* value < .05 was considered statistically significant.

An additional evaluation of total nerve gap was calculated as a sum of nerve damage length for proximal and distal nerve segments for each type of laceration and in situ measured length of the gap between transected nerve ends. The total nerve gap represents the gap after simulated resection of damaged nerve tissue. This evaluation was not statistically analyzed, as total nerve gap was calculated with group averages.

## Results

### Length of nerve damage

**Figure 7:**
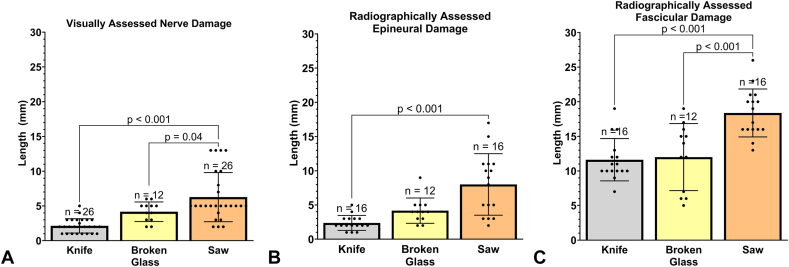
**A** Length of nerve damage measurements assessed visually, with pooled proximal and distal nerve segments and anatomical zones. Data analyzed using one-way ANOVA. **B** Radiographic measurement of epineural damage for each injury type with pooled nerve segments and anatomical zones. Data analyzed using Kruskal-Wallis. **C** Radiographic measurement of fascicular damage for each injury type with pooled nerve segments and anatomical zones. Data analyzed using one-way ANOVA. Data presented as mean ± SD.

Visually estimated length of damaged nerve increased with the complexity of laceration type from knife and glass to table saw, with significantly more damage occurring along the length of the nerve in table saw injuries (*P* = .04 and *P* < .001, respectively; Table 2 and Fig. 7A). Radiographically measured epineural damage was significantly longer in saw injuries than in knife injuries, *P* < .001 (Fig. 7B). Radiographically measured fascicular damage was significantly longer in the table saw than in the knife and broken glass lacerations (*P* < .001, Fig. 7C).

**Table 2:** Visually and Micro-CT Estimated Length of Nerve Damage by Injury Type, Data Pooled for Anatomical Zone and Nerve Segment

Evaluation Method	Statistic	Knife	Broken Glass	Table Saw
Visually assessed nerve damage (mm)	Mean ± SD (mm)	2.1 ± 1.0	4.2 ± 1.4	6.3 ± 3.5
	95% CI (mm)	1.7–2.6	3.3–5.1	4.8–7.7
Radiographic epineural damage (mm)	Mean ± SD (mm)	2.4 ± 1.1	4.2 ± 1.9	8.0 ± 4.5
	95% CI (mm)	1.8–3.0	3.0–5.3	5.6–10
Radiographic fascicular damage (mm)	Mean ± SD (mm)	11.6 ± 3.1	12.0 ± 4.8	18.4 ± 3.5
	95% CI (mm)	10.0–13.3	8.9–15.1	16.5–20.2

Epineural and fascicular nerve damage length showed no significant differences between anatomical zones (Table 3) or proximal versus distal nerve segments (Table 4), independent of the evaluation method.

**Table 3:** Length of Nerve Damage by Anatomical Zone, Data Pooled for Nerve Segment

Evaluation Method	Measurement	Knife		Broken Glass		Table Saw	
		Zone II	Zone V	Zone II	Zone V	Zone II	Zone V
Visually assessed nerve damage (mm)	n value	18	8	4	8	14	12
	Mean ± SD (mm)	2.0 ± 1.1	2.4 ± 0.9	2.5 ± 0.6	5.0 ± 0.8	4.4 ± 2.1	8.4 ± 3.8
	95% CI (mm)	1.5–2.6	1.6–3.1	1.6–3.4	4.4–5.6	3.2–5.6	6.0–10.8
Radiographic epineural damage (mm)	n value	8	8	8	4	8	8
	Mean ± SD (mm)	2.8 ± 1.0	2.0 ± 1.0	4.0 ± 2.3	4.5 ± 0.6	10.3 ± 4.7	5.6 ± 3.2
	95% CI (mm)	1.9–3.6	1.1–2.9	2.1–5.9	3.6–5.4	6.4–14.1	2.1–8.4
Radiographic fascicular damage (mm)	n value	8	8	8	4	8	8
	Mean ± SD (mm)	11.1 ± 3.5	12.1 ± 2.7	10.1 ± 4.6	15.8 ± 3.0	19.3 ± 4.0	17.5 ± 2.9
	95% CI (mm)	8.2–14.0	9.9–14.4	6.3–14.0	11.0–20.5	15.9–22.6	15.1–19.9

**Table 4:** Length of Nerve Damage by Proximal Versus Distal Nerve Segment, Data Pooled for Anatomical Zone

Evaluation Method		Knife		Broken Glass		Table Saw	
		Proximal	Distal	Proximal	Distal	Proximal	Distal
Visually assessed nerve damage (mm)	n value	13	13	6	6	13	13
	Mean ± SD (mm)	2.4 ± 1.2	1.9 ± 0.8	4.0 ± 0.9	4.3 ± 1.9	5.9 ± 3.5	6.7 ± 3.7
	95% CI (mm)	1.7–3.1	1.4–2.3	3.1–4.9	2.4–6.3	3.7–8.0	4.5–8.9
Radiographic epineural nerve damage (mm)	n value	8	8	6	6	8	8
	Mean ± SD (mm)	2.1 ± 0.8	2.6 ± 1.3	3.8 ± 1.2	4.5 ± 2.4	7.0 ± 4.9	9.0 ± 4.2
	95% CI (mm)	1.4–2.8	1.5–3.7	2.6–5.1	2.0–7.1	2.9–11.1	5.5–12.5
Radiographic fascicular damage (mm)	n value	8	8	6	6	8	8
	Mean ± SD (mm)	12.3 ± 3.5	11.0 ± 2.7	10.0 ± 4.2	14.0 ± 5.0	18.5 ± 2.1	18.3 ± 4.6
	95% CI (mm)	9.4–15.1	8.8–13.2	5.7–14.4	8.8–19.2	16.7–20.3	14.4–22.1

**Figure 8:**
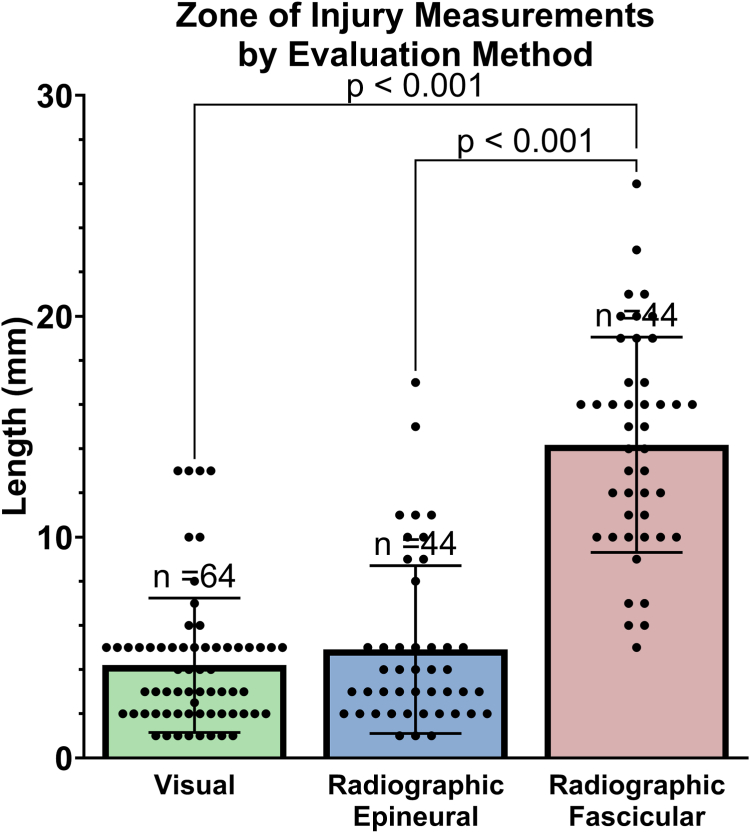
Comparison of visually estimated nerve damage length, radiographically measured epineural damage, and radiographically measured fascicular damage. Data analyzed using Kruskal-Wallis. Data presented as mean ± SD.

**Figure 9:**
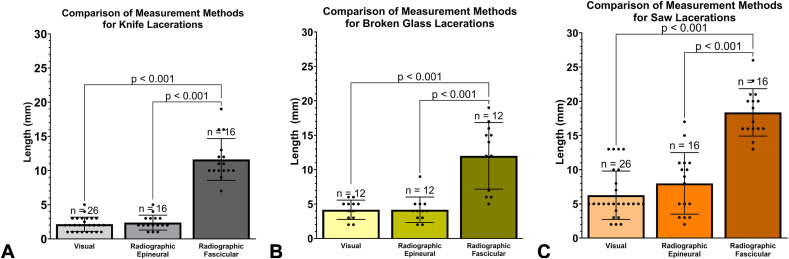
Comparison of visually estimated nerve damage length, radiographically measured epineural damage, and radiographically measured fascicular damage. **A** Knife lacerations, data analyzed using one-way ANOVA, **B** broken glass lacerations, data analyzed using Kruskal-Wallis, and **C** table saw lacerations, data analyzed using one-way ANOVA. Data presented as mean ± SD in all graphs.

Nerve damage was pooled for nerve segments, anatomical zones, and injury types for visual and radiographic measurement comparison. Visually estimated nerve damage length (4.2 ± 3.0 mm; 95% CI, 3.4–5.0 mm) was similar to radiographically measured epineural damage (4.9 ± 3.8 mm; 95% CI, 3.8–6.1 mm; Fig. 8); however, visually estimated nerve damage and radiographically measured epineural damage were significantly less extensive than radiographically measured fascicular damage (14.2 ± 4.9 mm; 95% CI, 12.7–15.7 mm), *P* < .001. This trend remained consistent independent of injury type, where radiographically measured fascicular damage was significantly more extensive than both radiographically measured epineural damage and visually estimated nerve damage length, *P* < .001 (Fig. 9A–C and Table 2).

### Nerve gap evaluation

**Figure 10:**
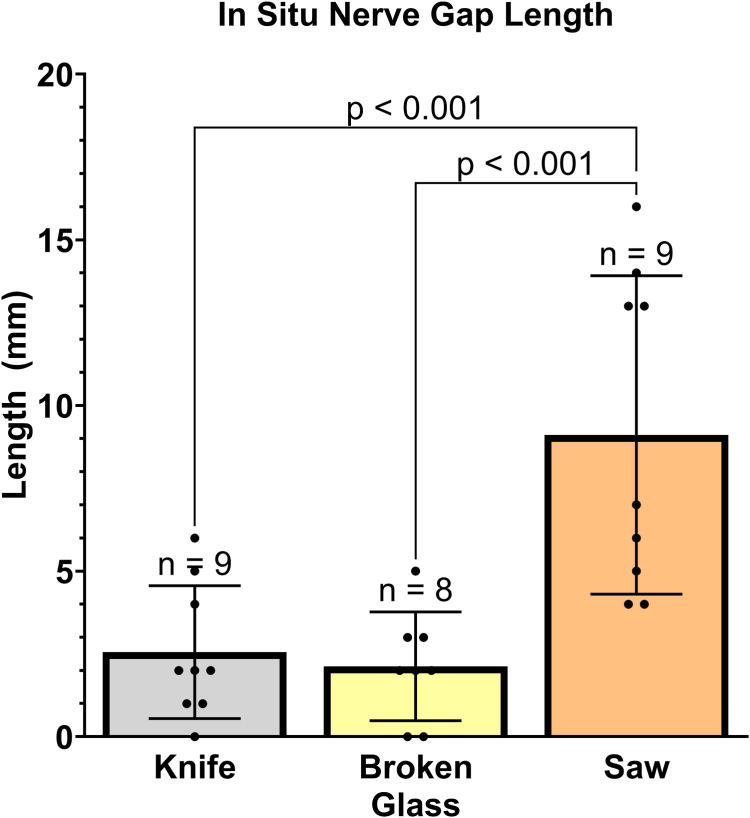
In situ nerve gap length between lacerated nerve ends. Data analyzed using one-way ANOVA, presented as mean ± SD.

Visual assessment of laceration sites showed that the most severe injuries were introduced with the table saw. The reported gap length showed that nerve injuries from the table saw (9.1 ± 4.8 mm; 95% CI, 5.4–13 mm) resulted in significantly longer nerve gaps than either the broken glass (2.1 ± 1.6 mm; 95% CI, 0.75–3.5 mm) or knife (2.6 ± 2.0 mm; 95% CI, 1.0–4.1 mm) injuries, *P* < .001 (Fig. 10). Additionally, there were no differences between anatomical zone II and V injuries.

### Total nerve gap

**Figure 11:**
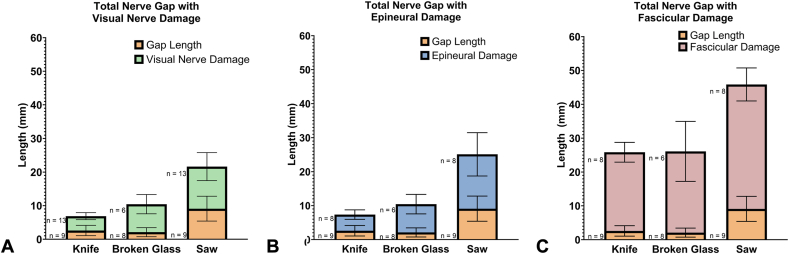
Measured in situ nerve gap plus length of nerve damage, showing the anticipated total nerve gap after resection of unhealthy tissue by **A** visual examination, **B** radiographic epineural measurement, and **C** radiographic fascicular measurement. Data presented as mean ± 95% CI.

The average total length of nerve damage for proximal and distal nerve segments for each type of laceration was added to the average measured in situ gap length, which represents the total estimated gap length that would result after resection of damaged nerve tissue, termed total nerve gap (Fig. 11A–C). These data averages showed that saw lacerations exhibited the largest potential total nerve gap (Table 5). The knife and broken glass showed notably less potential total nerve gap. These results were consistent across evaluation methods.

**Table 5:** Measured In Situ Nerve Gap Plus Length of Nerve Damage, Showing the Anticipated Total Nerve Gap After Resection

Evaluation Method	Statistic	Knife	Broken Glass	Table Saw
Visually assessed total nerve gap (mm)	Mean ± SD (mm)	4.3 ± 1.7	8.3 ± 2.7	12.5 ± 6.7
	95% CI (mm)	3.2–5.3	5.5–11.2	8.4–16.7
Radiographic epineural total nerve gap (mm)	Mean ± SD (mm)	4.8 ± 1.7	8.3 ± 2.8	16.0 ± 7.6
	95% CI (mm)	3.4–6.1	5.5–11.2	9.6–22.4
Radiographic fascicular total nerve gap (mm)	Mean ± SD (mm)	23.3 ± 3.5	24.0 ± 8.4	36.8 ± 5.8
	95% CI (mm)	20.3–26.2	15.1–32.9	31.9–41.6

## Discussion

The current study investigated differences in the extent of injury in a cadaver because of lacerations, evaluated visually and compared with extent of injury imaged by micro-CT. We hypothesized that high-resolution micro-CT would reveal a greater extent of damage than loupe visualization and assessment. This study highlights differences in extent of damage because of different injury types and the potential limitations of the tools currently used to evaluate nerve damage, including visual observation using loupes or surgical microscopes. We observed differences in nerve damage assessed with loupes versus micro-CT, which suggests that assessment using surgical loupe magnification was potentially insufficient for evaluating damage deep to the epineurium. Specifically, areas of “skip” lesions in nerve fascicles, which were present in lacerated nerve samples. Ideally, “skip” lesions would be verified with histology images; however, previous studies have used micro-CT to map 3-dimensional nerve structure, identify nerve fascicles, and differentiate fascicular tissue from other tissues.^8^ Although this limitation exists, less extensive damage was noted in loupe evaluations compared with micro-CT evaluations, which emphasizes the importance of adequately resecting damaged nerve until signs of healthy tissue are visible on the cross-sectional surface.

Visual assessment of the length of damage from the transected nerve end was similar to radiographically measured epineural damage, suggesting that surgeons accurately evaluated visible damage along the length of the nerve. However, visual estimations reported significantly less nerve damage than the radiographically measured fascicular damage in all laceration types. These differences were attributed to the inability to visualize damage deep to the epineurium, and the likelihood that the epineurium can appear normal even when the fascicles are damaged.^18^ This internal damage can only be evaluated by resecting additional nerve length to identify signs of healthy nerve tissue; however, these signs may not be readily apparent in acutely injured tissue.^4^

Visually estimated length of nerve damage increased with the complexity of laceration type from knife (mean, 2.1 mm) to broken glass (mean, 4.2 mm) to table saw (mean, 6.3 mm), with significantly less damage occurring in the knife and broken glass injuries compared with the table saw injuries. In radiographically evaluated epineural damage, the knife (mean, 2.4 mm) and broken glass (mean, 4.2 mm) created similar length of epineural damage, whereas the table saw (mean, 8.0 mm) created significantly greater lengths of epineural damage, independent of evaluation method. Saw injuries also resulted in the longest in situ nerve gap (mean, 9.1 mm). These results were similar to a previous animal study showing the most severe traumatic injury was caused by nerve avulsion and a sawing mechanism, whereas crushing and cutting mechanisms were less severe.^19^

The total gap length, which includes the in situ gap and length of nerve damage both proximal and distal to the laceration, was longest for table saw injuries. The mean total gap length of fascicular damage was up to approximately 26 mm in knife and broken glass injuries and approximately 46 mm in saw injuries, which would require repair with a nerve graft. It should be noted that both traumatic loss of tissue and nerve retraction may have contributed to the mean total gap length; however, the individual contributions of each are difficult to dispel, as tissue in a cadaveric model does not have the same properties as would be seen clinically. The extensive damage because of the saw mechanism was expected, as power tool injuries typically cause multiple levels of tissue damage including tendons and bones.^17^ However, the mean in situ nerve gap from knife injuries was larger than anticipated, as clean/sharp nerve transections are typically small gaps and have commonly been repaired with direct repair.^20^ The fascicular damage similarities across nerve segments and between anatomical zones suggest that nerve damage because of trauma was independent of location.

Although not necessarily predictive of outcomes after repair of lacerated nerves, findings provide insight into the structural integrity of the nerve beyond the cut surface. Our study showed variable degrees of intraneural damage with sharp transection injuries. The extent of nerve disruption was 3–4 times greater than what was visible on the outside for saw injuries and more limited with knife and glass and even multifocal with intervening areas of seemingly intact nerve. Fascicles were more sensitive to longitudinal damage from “skip” lesions than epineurium, suggesting that the epineurium adjacent to the cut ends may be preserved despite severe injury to the perineurium, the endoneurium, or both.^21^ Given the considerable internal damage, saw injuries were more comparable to avulsions than to sharp transections. It is difficult to discern the extent of internal damage after traumatic injury; therefore, in clinical practice, the surgeon should be ready to resect beyond the visible zone of injury and graft-repair or schedule a delayed surgical exploration to allow the zone of injury to demarcate. Resection of damaged nerve within the zone of injury is crucial to remove damaged tissue that may impair nerve regeneration and lead to neuroma formation.^6^

The current and practical use of optical magnification (loupes and/or operative microscope) may not be optimal for evaluating the extent of fascicular damage; however, the clinical implications of this damage are yet to be studied. Of note, the visual assessments performed in this study were limited to what was readily apparent without resecting nerve tissue, which was necessary to accurately perform the micro-CT imaging portion of the study. Although more extensive nerve damage was identified radiographically, the use of a micro-CT is not currently clinically feasible. High-resolution micro-CT is limited to evaluation of small ex vivo samples.^22^ Clinically, nerve damage is evaluated visually using loupes or a surgical microscope and by palpation. This could limit changes in clinical practice applied from findings in this study. Additionally, this study used cadaveric specimens for injuries, which do not have the same physiological tension as live tissues and may react to traumatic injury differently than live tissues. Also, traumatic injuries occur in uncontrolled circumstances; therefore, variations in injury severity are likely across all injury mechanisms.

In conclusion, this study used a novel high-resolution imaging method to characterize the extent of nerve damage resulting from common traumatic lacerations with a comparison to visual assessment of nerve damage. We found that the visual examination of length of nerve damage was significantly limited with loupe magnification alone, even with damage caused by a sharp knife. Our findings should encourage surgeons to suspect there might be more damage within the fascicles of the nerve than can be readily visualized. Although it is unknown if this damage will result in the formation of scar tissue, the existing body of evidence suggests that identifying and resecting this damaged tissue from the zone of injury is crucial to optimizing nerve repair outcomes, as damaged tissue may result in scar and scar tissue is known to impede nerve regeneration.^6^^,^^23^^,^^24^ The physiological and clinical implications of these findings will require further investigation and additional studies.

## Conflicts of Interest

Dr Zhukauskas is an employee of Axogen. Dr Smetana is a consultant for Axogen and is on the Axogen Advisory Board. Dr Strohl is on the Axogen Physician Advisory Board. Dr Wimalawansa has financial involvement/association with Axogen and CheckPoint Surgical. Dr Melamed is a consultant for Axogen. Dr Leversedge receives consulting fees from Axogen. Dr Marjoua is a consultant for Axogen. Dr Safa has financial involvement/association with Axogen. Dr Moore has no relevant conflicts of interest to disclose. This research was conducted with the assistance of Axogen Corporation. Bias in this study was controlled by a qualified radiologist verifying nerve damage measurements and statistical analysis of group differences.
