# Supplementary material for: More Than Epineurium Deep: Characterizing Peripheral Nerve Damage Using High-Resolution Micro-Computed Tomography for Simulated Peripheral Nerve Lacerations
Source: J Hand Surg Glob Online. 2025 Sep 18;7(6):100833. doi: 10.1016/j.jhsg.2025.100833 (PMC12481909; doi:10.1016/j.jhsg.2025.100833)
Supplement: Supplementary Table 1 [file mmc1.docx]

Table S1: Manuscript Definitions

| **Definition and description** | **Example Image** |
| --- | --- |
| ***In situ* evaluations** | |
| **Visually estimated length of damaged nerve:** Estimations of the nerve damage under examination using loupe magnification. Both the proximal and distal nerve stumps were examined, and estimated damage was recorded for the proximal and distal nerve stumps. This visual assessment was performed without trimming damaged nerve tissue. | 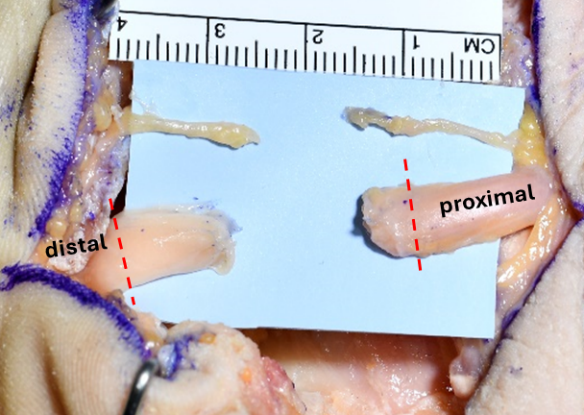 |
| **Post-injury in-situ gap length:**  After nerve laceration and neurolysis, surgeons measured the length between the proximal and distal nerve stump. This measurement was performed after surgeons evaluated the estimated length of nerve damage in each stump of the lacerated nerve. | 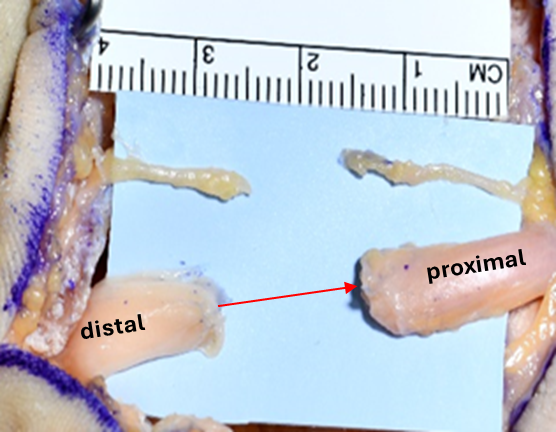 |
| **Nerve sample micro-CT image evaluations** | |
| **Radiographically measured epineural damage:**  measured using Dragonfly 3D software on radiograms and tomograms of nerve samples. It represents the length from the lacerated nerve end to where nerve fascicular area recovered, and intact epineurium was circumferentially identified. | 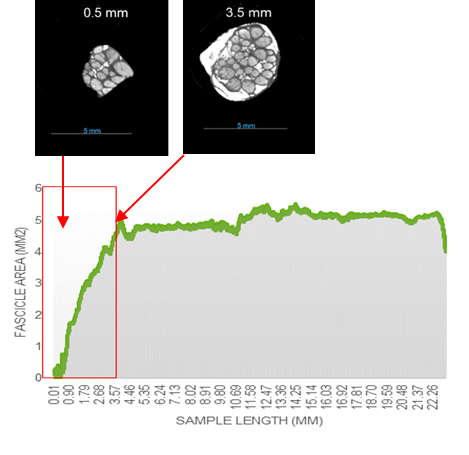  Nerve fascicular area over the length of sample curve slope (defined by red box) confirmed the level of fascicular area recovery. |
| **Radiographically measured fascicular damage:**  measured using Dragonfly 3D software on radiograms and tomograms of nerve samples. It represents the length from the lacerated nerve end through the adjacent tissues until the consistent fascicular radiopacity, indicative of healthy tissue was observed. | 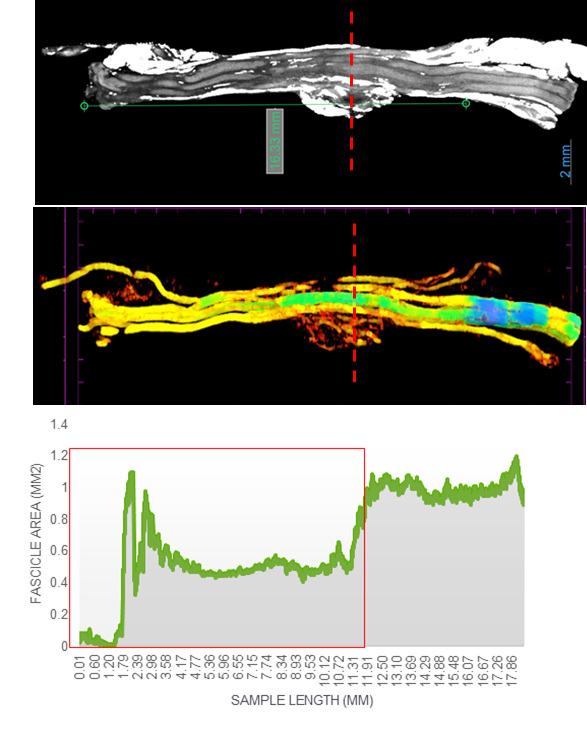  Nerve fascicular area over the length of sample curve confirmed the level of lower fascicular area within the damaged nerve tissue (defined by red box). |
| **“Skip” lesions:**  Radiographically identified features in the areas of fascicular damage. They were characterized by a sudden drop in the fascicular tissue radiodensity. Fascicular tissue radiodensity returned to normal passed the “skip” lesion. | 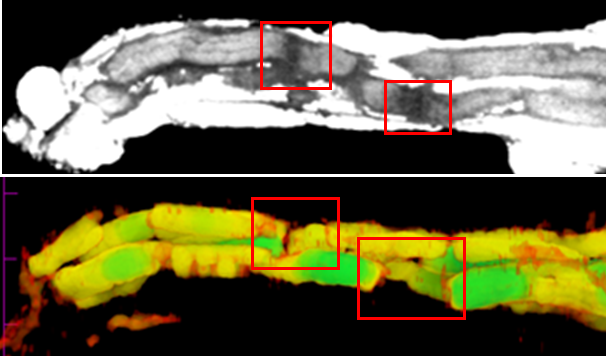 |
| **Total nerve gap:**  A calculated sum of three measurements: the length of **post-injury in-situ gap length** between lacerated nerve ends, the lengths of **radiographically measured epineural damage** in distal nerve and the lengths of **radiographically measured fascicular damage** in proximal nerve. | 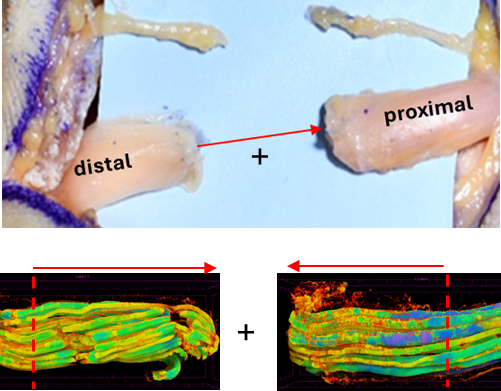 |
| **Debridement:**  Removal of damaged or fibrotic nerve tissue until uniform punctuated bleeding and pouching of the nerve fascicles is observed, and nerve tissue feels soft and pliable. |  |
| **Resection:**  A neurosurgical procedure in which a peripheral nerve is cut or removed. Also called neurectomy. |  |
| **Proximal nerve damage:**  (see radiographic fascicular damage); it represents the length of nerve tissue damage identified in the proximal to laceration nerve segment. |  |
| **Distal nerve damage:**  (see radiographic fascicular damage); it represents the length of nerve tissue damage identified in the distal to laceration nerve segment. |  |
